# Novel Convolutional Neural Network with Variational Information Bottleneck for P300 Detection

**DOI:** 10.3390/e23010039

**Published:** 2020-12-29

**Authors:** Hongpeng Liao, Jianwu Xu, Zhuliang Yu

**Affiliations:** 1 College of Automation Science and Technology, South China University of Technology, Guangzhou 510641, China; 201720116235@mail.scut.edu.cn; 2 Guangzhou Galaxy Thermal Energy Incorporated Company, Guangzhou 510220, China; gdxingchen@126.com; 3 Pazhou Lab., Guangzhou 510330, China

**Keywords:** variational information bottleneck, convolutional neural network, P300 signal detection

## Abstract

In the area of brain-computer interfaces (BCI), the detection of P300 is a very important technique and has a lot of applications. Although this problem has been studied for decades, it is still a tough problem in electroencephalography (EEG) signal processing owing to its high dimension features and low signal-to-noise ratio (SNR). Recently, neural networks, like conventional neural networks (CNN), has shown excellent performance on many applications. However, standard convolutional neural networks suffer from performance degradation on dealing with noisy data or data with too many redundant information. In this paper, we proposed a novel convolutional neural network with variational information bottleneck for P300 detection. Wiht the CNN architecture and information bottleneck, the proposed network termed P300-VIB-Net could remove the redundant information in data effectively. The experimental results on BCI competition data sets show that P300-VIB-Net achieves cutting-edge character recognition performance. Furthermore, the proposed model is capable of restricting the flow of irrelevant information adaptively in the network from perspective of information theory. The experimental results show that P300-VIB-Net is a promising tool for P300 detection.

## 1. Introduction

Brain-computer interface (BCI) provides a way for people to interact with the environment without any muscle activities, especially for people with amyotrophic lateral sclerosis, spinal cord injuries or other severe motor disabilities [1]. Event-related potentials (ERP), which is one of the important electroencephalography (EEG) signals, reflects neural activities after events. As a component of ERP, P300 is named after that positive potentials peaks occurs at about 300 ms after event-related stimuli [2]. P300 is widely used in BCI applications, like character recognition [3] and video surveillance [4].

Although P300 has been studied for long time, the detection of P300 is still challengeable in the case of low signal-to-noise ratio (SNR) due to unrelated neural activities and artifacts [5]. Lots of approaches were proposed for P300 detection [6,7,8,9]. Recently, the machine learning based methods achieved excellent performance on P300 detection [10,11,12]. For the traditional machine learning methods, feature extraction and classification are two of the key techniques. Principal component analysis [13], wavelet transform technique [14] were used for effective feature extraction. Support vector machine (SVM) is always used as a powerful classifier in P300 detection. In BCI Competition III [14], 17 SVM were ensembled for P300 detection and achieved the best performance. Group-sparse Bayesian linear discriminant analysis (gsBLDA) reached comparable classification accuracy, which treated signals of different channels as different groups [15].

Besides the traditional machine learning based methods, recently, deep learning models with different kinds of techniques had achieved great performance in many areas including the detection of ERP signal. A classic convolution neural network (CNN) for the detection of P300 waves was first proposed in Reference [16], that contains the spatial filter and temporal filter layers to well extract the spatial-temporal information of P300 signals. To make the CNN more robust, batch normalization and dropout were integrated into the proposed CNN, the resulting CNN is less sensitive to overfitting [17]. To further develop network which could find ERP components from data automatically, Restricted Boltzmann Machine (RBM) was utilized in ERP-Net [5]. The ERP-NET could discover all the ERP patterns contained in EEG signals. In addition, a spatial-temporal discriminative Restricted Boltzmann Machine (ST-DRBM) was further proposed [18] to learn spatial and temporal features separately and characterize the scalp distribution and temporal diversification. ST-DRBM has higher performance for ERP detection and it provides physiologically explainable results.

To further improve the robustness of network in P300 detection, in this paper, we propose a novel convolutional neural network based on variational information bottleneck. The proposed network, which is named as P300-VIB-Net, could reduce irrelevant information adaptively from the data. Hence, it is more robust against noise as well as irrelevant information. The contributions of this paper are summarized as: (1) A novel neural network architecture, P300-VIB-Net, is proposed for P300 detection. It combines the CNN as well as variational information bottleneck to make the network more robust against irrelevant information; (2) P300-VIB-Net reaches the state-of-art performance in P300 speller experiments; (3) We provide an explanation from the perspective of information theory on how the variational information bottleneck works with CNN. This also provides new insights on regularization technique.

## 2. Deep Learning Based on Variational Information Bottleneck

Deep neural networks can be explained in the information-theoretical framework [19] by information bottleneck (IB) that aims to find the short code for input which maintains the maximum information about output with mutual information [20]. The neural network with variational information bottleneck (VIB) show less overfitting and adversarial robustness [21]. Recently, variational discriminator bottleneck (VDB) with IB gets an important improvement in imitation learning, adversarial inverse reinforcement learning, and generative adversarial network (GAN) [22]. Information dropout was generalized by dropout based on IB, making better use of architectures with limited capacity [23]. In this section, an introduction on IB is presented as follows.

### 2.1. Information Bottleneck Principle

Relevant information in input data x∈X is defined as the information that signal x provides about output data y∈Y. Signal coding focuses on discovering the representation Z of X, as known as code or hidden variables, keeping the most information about Y, which is measured by mutual information I(Z,Y) between Z and Y

(1) $$I\left(Z,Y\right)=\int_{z}\int_{y}p\left(z,y\right)\log\frac{p(z,y)}{p(y)p(z)}dydz.$$

It is obvious that I(Z,Y) achieves the maximal value by taking the identity coding of input data as Z=X. This identity encoding is not a useful representation of the processed data. Hence, in practice, the constraint $I\left(X,Z\right)\leq I_{c}$ is imposed as the ‘bottleneck’, where $I_{c}$ is a constant, restricting the information from X to Z. This suggests the objective

(2) $$\max\;I\left(Z,Y\right)\;s.t.\;\;I\left(X,Z\right)\leq I_{c}.$$

By introducing a Lagrange multiplier β, the above problem can be formulated as minimizing the function below to get the ideal representation,

(3) −I(Z,Y)+βI(X,Z).

Minimizing the first term enhances the transfer of information from the intermediate coding variable Z to output variable Y, while minimizing the second term limits the transfer of information from the input variable X to the intermediate coding variable Z. We can find a suitable β to preserve minimal information from X to Z and the information in Z is sufficient to predict Y.

The information bottleneck principle discussed above defines an optimal representation and holds the most distinctive information about Y in X. However, the computation about mutual information in information bottleneck principle is always hard except some very restrictive cases. How to simplify the calculation of problem in (3) is always an important problem in practice.

### 2.2. Variational Information Bottleneck

To solve the computational problem in IB, two significant improvements were proposed in the variational information bottleneck [21]. Firstly, variational inference is applied to build an upper bound of the function of IB. Secondly, the objective function can be optimized by stochastic gradient descent with the reparameterization trick [24]. Deep neural networks can be used for parameterization of distributions.

For I(Z,Y) defined in (1), since p(y|z) is difficult to obtain in practice, we use a variational approximation q(y|z) to approximate p(y|z). Since the Kullback-Leibler divergence is non-negative, that is, KL(p(y|z)||q(y|z))≥0, we have

(4) $$\begin{array}{c} \int_{y}p\left(y|z\right)\log p\left(y|z\right)dy\geq \int_{y}p\left(y|z\right)\log q\left(y|z\right)dy. \end{array}$$

Therefore,

(5) $$\begin{array}{ccc} I(Z,Y) & & =\int_{z}p\left(z\right)\int_{y}p\left(y|z\right)\log\frac{p(y|z)}{p(y)}dydz \end{array}$$

(6) $$\begin{array}{ccc} & & \geq \int_{z}p\left(z\right)\int_{y}p\left(y|z\right)\log\frac{q(y|z)}{p(y)}dydz \end{array}$$

(7) $$\begin{array}{ccc} & & =\int_{z}\int_{y}p\left(z,y\right)\log q\left(y|z\right)dydz+H\left(Y\right), \end{array}$$

where $H\left(Y\right)=-\int_{z}p\left(z\right)\log p\left(z\right)dz$ is independent of the optimization, and it can be neglected. Recall Markov assumption about joint distribution p(X,Y,Z) in Reference [21], which is p(Z|X,Y)=p(Z|X), corresponding to the Markov chain Y↔X↔Z, we have $p\left(z,y\right)=\int_{x}p\left(x\right)p\left(y|x\right)p\left(z|x\right)dx$. Consequently,

(8) $$\begin{array}{c} I\left(Z,Y\right)\geq \int_{z}\int_{y}\int_{x}p\left(x\right)p\left(y|x\right)p\left(z|x\right)\log q\left(y|z\right)dxdydz. \end{array}$$

Similarly, we can get the upper bound I(X,Z) of the second term in (3), because of KL(p(z)||a(z))≥0, where a(z) is the variational approximation of p(z), which is

(9) $$\begin{array}{c} \int_{z}p\left(z\right)\log p\left(z\right)dz\geq \int_{z}p\left(z\right)\log a\left(z\right)dz. \end{array}$$

Therefore,

(10) $$\begin{array}{cc} I(X,Z) & =\int_{z}\int_{x}p\left(z,x\right)\log\frac{p(x,z)}{p(x)p(z)}dxdz \end{array}$$

(11) $$\begin{array}{ccc} & & =\int_{z}\int_{x}p\left(z|x\right)p\left(x\right)\log\frac{p(z|x)}{p(z)}dxdz \end{array}$$

(12) $$\begin{array}{ccc} & & \leq \int_{x}p\left(x\right)KL\left(p\left(z|x\right)||a\left(z\right)\right)dx. \end{array}$$

Using empirical data distribution $p\left(x,y\right)=\frac{1}{N}\sum_{n=1}^{N}\delta_{x_{n}}\left(x\right)\delta_{y_{n}}\left(y\right)$, where *N* is the number of samples, we can write the upper bound as

(13) $$\begin{array}{cc} \frac{1}{N}\sum_{n=1}^{N} & [-\int_{z}p\left(z|x_{n}\right)\log q\left(y_{n}|z\right)dz\\ & +\beta \mathrm{KL}(p\left(z|x_{n}\right)||a\left(z\right))]. \end{array}$$

The first term is in the form of a cross-entropy loss function. The second term can be regarded as a regularization term. a(z) is the distribution we assume, usually a standard normal distribution. p(z|x) is an encoder, which transforms X into Z. Suppose the encoder is of the form $p\left(z|x\right)=N(z|f_{e}^{\mu }\left(x\right),f_{e}^{\Sigma }\left(x\right))$, where $f_{e}$ is a neural network which outputs both the mean μ and covariance matrix Σ, we can use reparameterization trick to generate z=f(x,ϵ) which is a deterministic function of x and Guassian random variable ϵ. Since the noise ϵ is independent of parameters of the model, it is easy to take gradients in the training process. If our choice of p(z|x) and a(z) allows computation of an analytic Kullback-Leibler divergence, we can get further simplified objective function in training.

### 2.3. P300-VIB-Net

**Figure 1:**
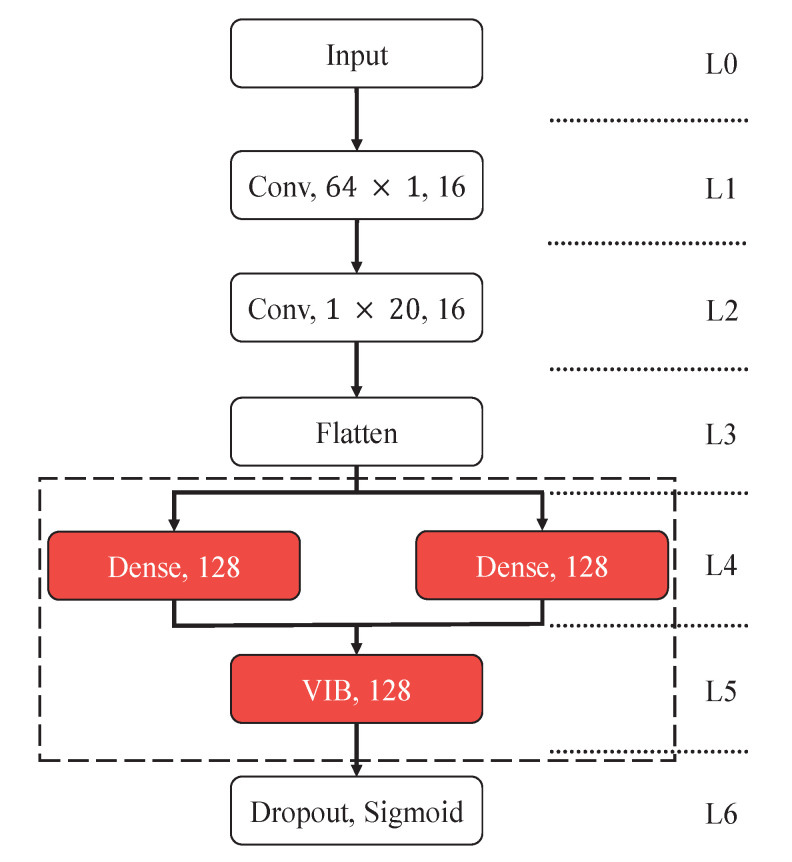
Details of network architecture. Pivotal information of each layer is shown in solid boxes. Crucial part of the proposed model is shown in dashed box.

In this paper, we proposed a new neural network for EEG classification. The proposed network as shown in Figure 1 is based on VIB and classic convolutional network architecture which is widely used in P300 detection problem. Parameters in the middle of convolution layers represents kernel size and parameter at the end of convolution layers is the number of kernels. The first few layers L0, L1, L2, and L3 of the model are similar to the layers of traditional convolutional network for P300 detection. L0 is the input layer. The size of input data to L0 is the number of channels multiplied by the length of signals in the time domain. L1 plays a role as a spatial filter to get the best combinations of signals from all electrodes. L2 serves as a temporal filter as well as a sub-sampler, which extracts the most important time-domain features. In L3, the feature map matrices, which are the outputs of L2, are flattened into vectors and input to the following fully connected layers.

In L4, there are two different fully connected networks that take the output of L3 as input to generate the mean and variance of encoder p(z|x) as

(14) $$\begin{array}{c} p(z|x)=N(z;\mu ,e^{\hat{\sigma }}) \end{array}$$

(15) $$\begin{array}{c} \left(\mu ,\hat{\sigma }\right)={\mathrm{NeuralNet}}_{\phi },\left(x\right) \end{array}$$

where ${\mathrm{NeuralNet}}_{\phi }\left(x\right)$ represents the layers L0-L1-L2-L3-L4, ϕ is parameters of the network layers, μ and $\hat{\sigma }$ correspond to the output of two fully connected networks in L4. In order to guarantee the non-negativeness of covariance, the exponential of $\hat{\sigma }$ is used to represent the variance of z.

In L5, reparameterization tricks is applied for easy calculating of gradients. Firstly, we produce ϵ by standard normal distribution function. Then, we generate z with μ, $\hat{\sigma }$ and ϵ as

(16) $$\begin{array}{c} \epsilon ∼N(0,I) \end{array}$$

(17) $$\begin{array}{c} z=\mu +e^{\hat{\sigma }}⊙\epsilon . \end{array}$$

In L6, in order to avoid overfitting, the dropout [25] is used. Dropout is a universally used technique in deep learning, which makes the existence of any particular hidden unit untrustworthy and cuts down the co-adaptation of neurons, alleviating overfitting in neural networks at the cost of increased training time. By generating a binary vector r whose elements follow Bernoulli distribution with *p* as the parameter representing the means drop rate in dropout, we have the output as

(18) $$\begin{array}{c} r∼\mathrm{Bernoulli}(p) \end{array}$$

(19) $$\begin{array}{c} y=f(r*z+b), \end{array}$$

where *f* is the sigmoid function $f\left(x\right)=\frac{1}{1+e^{-x}}$ that represents the P300 signal detection probability.

In the P300-VIB-Net, p(z|x) is parameterized by L0-L1-L2-L3-L4-L5, which encodes X into intermediate representation Z. We suppose that a(z) is N(0,I), q(y|z) is parameterized by L6 as described in (19). After taking the analytical result of KL divergence as [24], the loss function can be formulated as

(20) $$\begin{array}{cc} \mathrm{Loss}= & \frac{1}{N}\sum_{n=1}^{N}-\int_{z}p\left(z|x_{n}\right)\log q\left(y_{n}|z\right)dz\\ & -\frac{\beta }{2}\sum_{j=1}^{J}\left[1+log\left({\left(\sigma_{j}\right)}^{2}\right)-{\left(\mu_{j}\right)}^{2}-{\left(\sigma_{j}\right)}^{2}\right], \end{array}$$

where *J* is the size of hidden variable z, $\mu_{j}$ and $\sigma_{j}$ are j-th elements to generate z. $y_{n}$ are labels given in the datasets. The cross entropy of probabilities y and labels $y_{n}$ will be calculated during the learning process to optimize (20).

## 3. Experimental Results

In this section, the experimental results of the proposed network on P300 speller paradigm will be presented. The proposed model, P300-VIB-Net, will be compared with other state-of-art algorithms to show the effectiveness of the proposed method.

### 3.1. P300 Speller Paradigm

The occurrence of P300 is related to the human’s reaction to the stimulus. P300 is relatively obvious and easy to observe among all ERP components. Thus, P300 is considered to reflect the process of receiving stimulation. Subjects usually are shown with a random sequence of target and non-target stimuli based on the oddball paradigm. Generally, the smaller the probability of the appearance of the target stimulus, the greater the magnitude of P300, from which we can find the target that subjects focus on.

**Figure 2:**
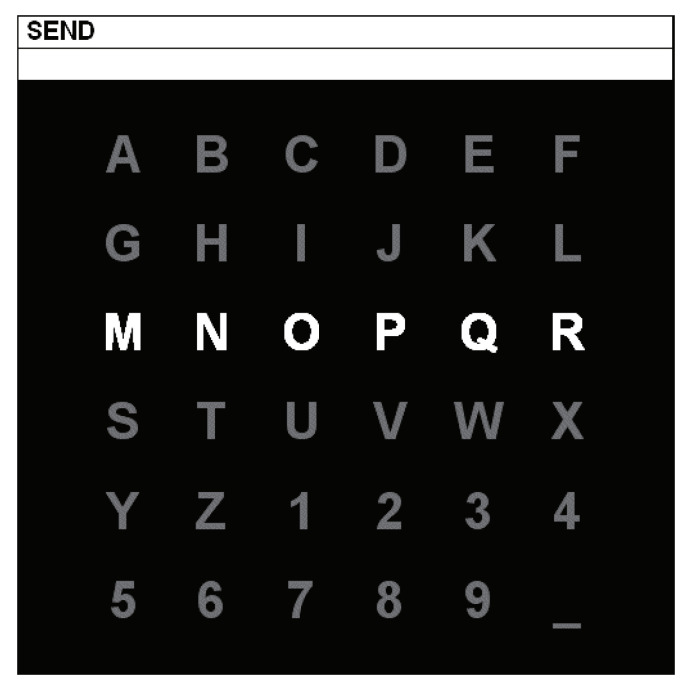
P300 speller interface in brain-computer interface (BCI) Competition III [26].

Data set II of BCI competition III which is widely used as a benchmark data set for P300 detection is used as the test data set. The P300 speller paradigm of data set II was described in Reference [26]. It was based on the principle that flashing characters on the screen that subjects focus on will stimulate the presence of P300. The stimulation graphical user interface consists a 6×6 matrix, including 36 characters, as shown in Figure 2. Subjects are asked to focus on one character given in a prompter on top of the matrix. Every row and every column of the matrix flash once at the rate of 5.7 Hz randomly in each epoch. There are 2 of 12 intensifications that contain the target character at the intersection of the row and the column. Therefore, the target character can be detected by distinguishing P300 and non-P300 signals of every flashing. In other words, the classification of 36 classes is transformed into binary classification problem. 30 P300 and 150 non-P300 signals are obtained after repeating 15 times for each character. There are 85 characters in the training set and 100 characters in the test set of subject A and subject B.

### 3.2. Data Preprocessing

The 64 channel EEG data was collected with sampling rate 240 Hz. Data preprocessing consists four steps. First of all, the time window is chosen as 0∼670 ms after flashing. Each sample is of size 64×160. Secondly, data is bandpass filtered by a 4-th order Chebyshev type I filter with bandwidth 0.1–20 Hz. Thirdly, every sample is normalized to be zero mean and unit variance. Last but not the least, since data in training sets and test sets is unbalanced, we duplicate the P300 signals 4 times to keep the data sets balance.

### 3.3. Experiments of P300-VIB-Net

The network model used in experiments is shown in Figure 1. Compared to traditional CNN network for P300 detection, the most significant modification is layers L4 and L5, which are the kernel components of VIB network presented in the dashed box.

We can get the probability $y_{i}$ by P300-VIB-Net to determine whether the signal contains P300 component when one row or one column flashes. We can get the coordinates of target character by

(21) $$\begin{array}{c} i_{x}=\arg\max_{1\leq i\leq 6}y_{i} \end{array}$$

(22) $$\begin{array}{c} i_{y}=\arg\max_{7\leq i\leq 12}y_{i}, \end{array}$$

where *i* is the index of row or column range in [1,12], $y_{i}$ represents the probability that the signal is P300 while the *i*th row or column in the matrix is intensificated, $i_{x}$ and $i_{y}$ represent the row and column index with most likely P300 signals. The target character is the one at the intersection of $i_{x}$-th row and $i_{y}$-th column.

In Table 1, the character recognition rate of subject A and B with 1, 5, 10, and 15 epochs are presented. The results of P300-VIB-Net and other models including E-SVM, CNN, MCNN-1, BN3, ERP-Net, PST-DRBM, SAE-ESVM, CM-CW-CNN-ESVM, are presented for comparison. There are two algorithms that combine traditional machine learning and deep learning and achieve impressive recognition performance. Sparse autoencoder (SAE) is used for deep feature extraction and ESVM is used for classification in SAE-ESVM. While in CM-CW-CNN-ESVM, high-level features are extracted by CNN. After that, Fisher ratio (F-ratio) is used to select these features to get optimal features. However, when the number of repeat epochs is relatively small, BN3, PST-DRBM, and ERP-Net achieve the best performance in terms of average character recognition rate. With increasing number of repeat epochs, the character recognition rate of P300-VIB-Net becomes the highest one.

### 3.4. The Role of VIB Term

As mentioned above, the VIB term represents the mutual information between the input data and intermediate code. Minimizing the VIB term means restricting the information from input data X to intermediate representation Z as well as output variable Y. Maximization the cross-entropy between Z and Y will force the information flowing from input to predict output. Therefore, when these two processes work simultaneously, the whole loss makes the model focus only on information that is related to output in input data. The resulting model is less disturbed by information that is not related to the output. Hence, VIB could improve the generalization performance. This can be verified by finding the relationship between β and character recognition rate.

**Figure 3:**
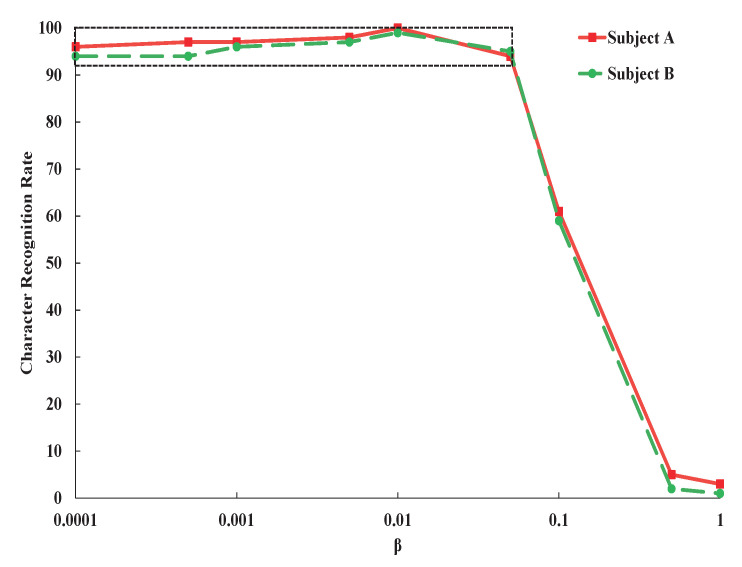
Variation of character recognition rate of subject A and subject B with the changing β of variational information bottleneck (VIB) regularization term.

**Figure 4:**
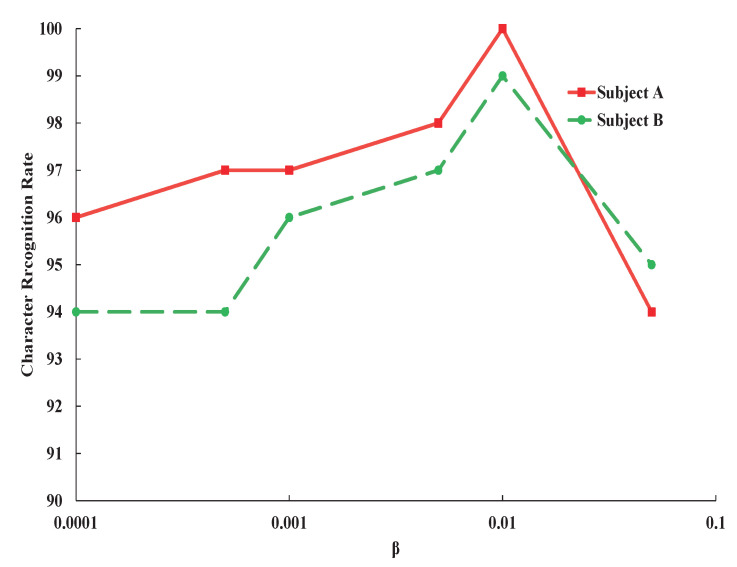
Variation of character recognition rate of subject A and subject B with the weight β changing around optimal value.

The results in Figure 3 and Figure 4 are obtained by manually adjusting β to show the effects of β on the character recognition rate. As shown in Figure 3, the most suitable value of β is 0.01, which make the network attains the best character recognition rate. When increasing β larger than 0.01, the character recognition rate is gradually reduced. This can be explained that with bigger β, more and more information including discriminative information are blocked from input to intermediate code (feature). With extremely large value of β, the information are totally blocked and the character recognition totally failed.

To present a more clear illustration, the curve in the dashed box in Figure 3 is magnified and shown in Figure 4. When we increase β from 0.0001 to 0.01, the character recognition rate increases, which indicates that restriction of mutual information between input signals *X* and code *Z* could improve the performance of character recognition by blocking label irrelevant information from input data to feature vector. From these results, we could find that whether the weight β is too large or too small, the classification performance will seriously be degraded.

## 4. Conclusions

Event-related potentials detection is an important problem in BCI research. The low signal-to-noise ratio of EEG signal makes the detection of ERP challengeable. A novel convolutional neural network based on VIB is proposed for P300 detection in this paper. With VIB regularization term added to the traditional cross-entropy loss, the information flowing from input data to intermediate code could be controlled and the label irrelevant information is removed from intermediate variables (features). The experimental results demonstrate that P300-VIB-Net could achieve state-of-art performance in the P300 speller character recognition problem. VIB constraint in P300-VIB-Net enhances the generalization performance of the model. On the other hand, the performance of P300-VIB-Net will deteriorate when the amount of data is relatively small, because it’s difficult to estimate information with small amount of data. In our future work, we will explore models based on VIB with other problems in BCI. 

## Figures and Tables

**Table 1:** Character recognition rate of different models.

Subjects	Epochs	Models								
		CNN [16]	MCNN-1 [16]	E-SVM [14]	BN3 [17]	PST- DRBM [18]	ERP-Net [5]	SAE- ESVM [27]	CM-CW-CNN- ESVM [28]	Proposed
A	1	16	18	16	22	**24**	22	21	22	15
	5	61	61	72	73	**75**	**75**	72	64	66
	10	86	79	83	86	**90**	**90**	**90**	86	88
	15	97	97	97	98	98	99	**100**	99	**100**
B	1	35	39	35	**47**	43	42	40	37	32
	5	79	77	75	76	79	77	**80**	**80**	72
	10	91	92	91	95	94	**96**	90	95	94
	15	92	94	96	95	98	98	98	**99**	**99**
Avg	1	25.5	28.5	25.5	**34.5**	33.5	32	30.5	29.5	23.5
	5	70	69	73.5	74.5	**77**	76	76	72	69
	10	88.5	85.5	87	90.5	92	**93**	90	90.5	91
	15	94.5	95.5	96.5	96.5	98	98.5	99	99	**99.5**

## Data Availability

Publicly available datasets were analyzed in this study. This data can be found here: http://www.bbci.de/competition/ii/#datasets.
